# Stigma toward Wuhan people during the COVID-19 epidemic: an exploratory study based on social media

**DOI:** 10.1186/s12889-021-12001-2

**Published:** 2021-10-29

**Authors:** Yazheng Di, Ang Li, He Li, Peijing Wu, Simin Yang, Meng Zhu, Tingshao Zhu, Xiaoqian Liu

**Affiliations:** 1 CAS Key Laboratory of Behavioral Science, Institute of Psychology, Beijing, 100101 China; 2 Department of Psychology, University of Chinese Academy of Sciences, Beijing, 100049 China; 3 Department of Psychology, Beijing Forestry University, Beijing, 100083 China; 4 Department of Psychology, Beijing Normal University, Beijing, 100875 China; 5 Hubei University of Economics, Wuhan, 430205 China

**Keywords:** Stigma, COVID-19, Infectious disease, Social media, Weibo

## Abstract

**Background:**

Stigma associated with infectious diseases is common and causes various negative effects on stigmatized people. With Wuhan as the center of the COVID-19 outbreak in China, its people were likely to be the target of stigmatization. To evaluate the severity of stigmatization toward Wuhan people and provide necessary information for stigma mitigation, this study aimed to identify the stigmatizing attitudes toward Wuhan people and trace their changes as COVID-19 progresses in China by analyzing related posts on social media.

**Methods:**

We collected 19,780 Weibo posts containing the keyword ‘Wuhan people’ and performed a content analysis to identify stigmatizing attitudes in the posts. Then, we divided our observation time into three periods and performed repeated-measures ANOVA to compare the differences in attitudes during the three periods.

**Results:**

The results showed that stigma was mild, with 2.46% of related posts being stigmatizing. The percentages of stigmatizing posts differed significantly during the three periods. The percentages of ‘Infectious’ posts and ‘Stupid’ posts were significantly different for the three periods. The percentage of ‘Irresponsible’ posts was not significantly different for the three periods. After government interventions, stigma did not decrease significantly, and stigma with the ‘Infectious’ attitude even increased. It was not until the government interventions took effect that stigma significantly reduced.

**Conclusions:**

This study found that stigma toward Wuhan people included diverse attitudes and changed at different periods. After government interventions but before they took effect, stigma with the ‘Infectious’ attitude increased. After government interventions took effect, general stigma and stigmas with ‘Infectious’ and ‘Stupid’ attitudes decreased. This study constituted an important endeavor to understand the stigma toward Wuhan people in China during the COVID-19 epidemic. Implications for stigma reduction and improvement of the public’s perception during different periods of epidemic control are discussed.

## Background

Emerging infectious diseases may lead to stigmatization [1] toward patients and those living in epidemic-stricken areas, which has various negative effects on target groups [2–4]. First, individuals suffering from stigma are at increased risks of mental and physical health problems, such as depression, hypertension, coronary heart diseases, and stroke, as well as damaged social wellbeing [1, 5–7]. Second, stigmatized patients may avoid seeking health care, which creates difficulties for public health authorities in controlling a disease [8]. Third, if people avoid areas related to the disease, stigma can cause considerable economic losses [3, 9].

At the end of 2019, the first COVID-19 patient from China was diagnosed in Wuhan. Afterward, the disease spread rapidly across China, which had a huge impact on society. Evidence and concerns about the social stigma associated with COVID-19 were raised [10–14]. To date, most of the studies have focused on the perceived stigma of healthcare workers [15–18] and COVID-19 survivors [19, 20]. These studies indicated a severe level of stigmatization. However, few studies have focused on how social stigma increases during the early stage of the disease. Moreover, while these studies mainly focused on the stigma toward Asians or Chinese people [10, 21], there was no empirical research on social stigma toward Wuhan people in China. As a group living in the place where the disease first broke out, social stigma in China may influence many aspects of the lives of Wuhan people. Therefore, understanding and mitigating the social stigma in China toward Wuhan people are necessary.

Identifying the specific attitudes associated with stigma is an important step for stigma mitigation [22, 23]. Traditional strategies aimed at reducing social stigma include information-based approaches, behavior interventions, and contact with affected groups [24]. Information-based approaches are widely used in public health interventions for reducing stigma, while behavioral interventions and contact are usually used in specifically targeted groups [25]. Studies have shown that information-based approaches that target specific corresponding attitudes are more effective at changing stigma than traditional public education programs that focus only on the propaganda of knowledge [24, 26]. For example, a common stigmatizing attitude toward infected individuals is to blame them for spreading the disease. In this case, education about factual knowledge plays little role in changing this stigmatizing attitude, and target-specific strategies should be intervention in their biased attribution [27]. In summary, anti-stigma campaigns should go beyond factual knowledge to strategically address specific public attitudes [25].

According to the stigma communication model [28, 29], four categories of message content, namely, mark, group labeling, responsibility, and peril, play vital roles in shaping stigma. Mark and labels are cues and descriptions that identify and separate the stigmatized group. When COVID-19 is labeled as the Wuhan virus, stigma is created toward those who are living in Wuhan, passed through Wuhan, or have a local accent [10]. They are labeled with negative characteristics. Responsibility refers to blaming by making attributions about a person’s choice and control. Travelers from Wuhan may be regarded as choosing to travel during COVID-19 and hence should bear responsibility for the outbreak. Peril connects marked, labeled, and responsible persons to physical or social harm to the community’s well-being. As a result, Wuhan people may be regarded as a threat to other areas in China [21].

An increasing number of researchers have considered stigma to change over the course of a single illness [30–33]. The social context is changing as the epidemic matures, and knowing about changes in stigma over time is essential for researchers to design mitigation strategies targeted at the social context [32]. However, such studies mainly focused on small groups [31, 34, 35], which were limited by the lack of representativeness. Studies that included large groups typically measured stigma changes over long-term and fixed intervals (e.g., several months or years) [32, 33]. In terms of emerging infectious diseases, public attitudes may rapidly change with the progression of an epidemic [36]. Such long-term intervals are insufficient to describe the fine-grained dynamics of stigma. In addition, most of the existing studies relied on retrospective investigations conducted a long time after the disease outbreak, and their conclusions were discredited because of potential recall bias [33, 34]. To explore the detailed changes of the stigma associated with emerging infectious diseases, a new data collection and analytical method is necessary.

In recent years, online social networks (OSNs) have become an increasingly popular approach for analyzing stigma and associated attitudes [37–39]. OSNs (e.g., Weibo, Twitter) provide chances to comprehensively understand the psychological state of participants in a noninvasive way [40]. Additionally, using records of user behavior on OSNs can avoid recall bias. From Weibo, the most popular Chinese microblogging service provider in China, researchers can collect a large number of timely samples and analyze fine-grained stigma changes [41], improving the representativeness and time sensitivity of the findings.

Specifically, this study examined stigma toward Wuhan people using data extracted from Weibo during COVID-19 (from December 1, 2019, to April 18, 2020). By identifying stigmatizing posts, we ascertained the prevailing stigmatizing attitudes and traced their changes during the epidemic in China. The results provide important information for mitigating the stigma toward people in epidemic center areas.

## Methods

### Data collection

The samples were from the Weibo data pool [42] containing 1.16 million active users. The retrieved data included user profiles, network behaviors (e.g., forward, reply), and post contents. In this study, we first collected Weibo posts (microblogs) from the data pool by the following criteria:

1. Published from December 1, 2019 to April 18, 2020.
2. Containing the keywords ‘Wuhan People’(“武汉人”).

We decided the time because the disease was first identified in December 2019 in Wuhan and the thoroughfare leaving Wuhan was reopened on April 8.

### Coding

After data collection, human coders performed content analysis to identify stigmatizing expressions in Weibo posts.

**Fig. 1:**
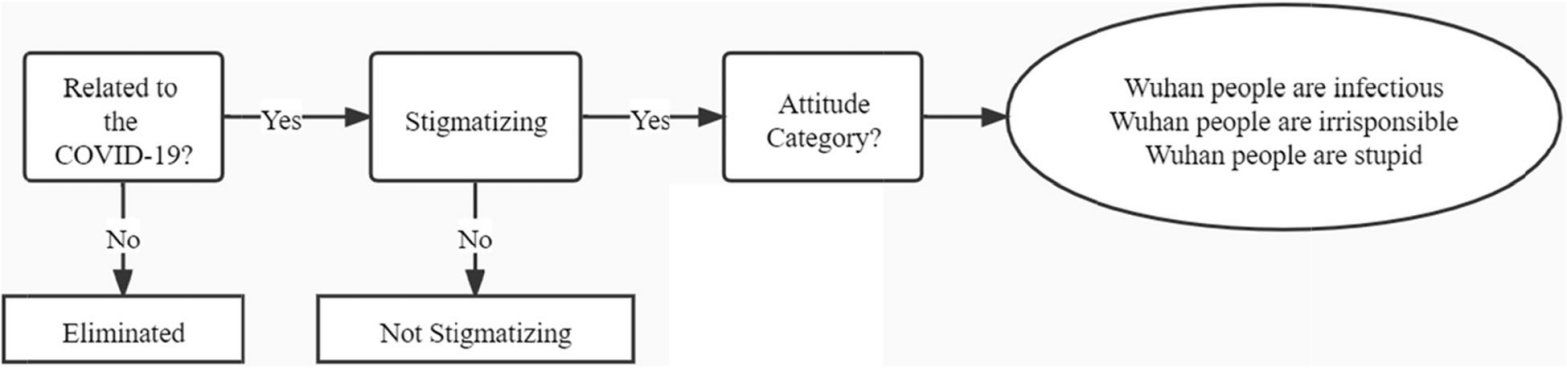
The coding process

The coding framework was built based on evidence and consensus. Specifically, the setting of the coding framework first referred to previous research [2, 43]. After browsing all the content on Weibo, the researchers summarized several categories of stigmatizing attitudes: infectious, irresponsible, and stupid (see Table 1). The whole process of coding work can be found in Fig. 1. First, the coders judged whether a post was related to COVID-19 and whether the post was stigmatizing. If so, the coders were to assign the post into one of the stigmatizing attitude categories. The detailed criteria for judgment are provided in the Supplementary Materials.

**Table 1:** Coding framework for stigmatizing content

Stigma Type	Definition	Example
Infectious	Wuhan people are infectious.	People in Wuhan now are mobile viruses. I suggest all the Wuhan people should be isolated.
Irresponsible	The spread of the virus or the spread of the epidemic is due to the irresponsibility of the Wuhan people.	If the Wuhan people had taken their responsibility and stopped going everywhere, the epidemic would be better than now!
Stupid	The outbreak was caused by the ignorance and stupidity of the Wuhan people, or it was caused by some backward or uncivilized habits of the Wuhan people.	I used to say that the Cantonese people are barbaric to eat all kinds of wild animals, but now it seems that the Wuhan people have a better appetite than Cantonese.

Four graduate students majoring in psychology took part in the study. First, the coders were trained on a small sample of 500 posts that were randomly selected from the total posts to reach substantial consistency on the training sample. Second, they analyzed the total posts, and each analyzed a different subset of the total posts.

### Data elimination

Before statistical analysis, we identified the users who posted related posts as well as their geo-location and gender. Further analysis focused on the epidemic and was based on the provincial area in China, so we eliminated posts according to the following criteria:

1. Not related to the COVID-19 according to the human coders.
2. Without user location information or with users located overseas

### Statistics

First, we performed an interrater reliability analysis to assess the degree to which coders consistently recognized stigmatizing Weibo posts and identified specific attitudes of stigma in the study. We calculated Light’s kappa coefficient [44] using Siegel & Castellan’s variant [45] for the coding results of the 4 coders on the training sample. Siegel & Castellan’s variant is used to correct bias problems of the coding results [46].

To analyze the changes in stigma over time, we calculated daily percentages of stigmatizing posts in total posts and drew a picture of their time trends. Furthermore, we divided our observation time into 3 periods as follows:

1. Period 1 was before government interventions (pre-GI) from Dec 31, 2019, to Jan 22, 2020. On Dec 31, 2019, Wuhan Centers for Disease Control notified the cases of COVID-19 for the first time. The number of confirmed cases began to rise, but the government did not adopt strict isolation measures until Jan 23, 2020.
2. Period 2 was the period after government interventions but before they took effect (post-GI). It was from Jan 23, 2020, to Feb 20, 2020. On Jan 23, 2020, the government began to take strict measures to control the disease, including a lockdown of Wuhan city and home quarantine orders. However, the increase in confirmed cases sped up because it took time for the interventions to take effect.
3. Period 3 was after government intervention took effect (eff-GI). It was from Feb 21, 2020 to Apr 8, 2020. On Feb 21, the Chinese government issued official documents to promote the resumption of work and production [47], indicating effective control of the local epidemic. During this period, the increase in confirmed cases slowed down. On Apr 8, 2020, the thoroughfare leaving Wuhan was reopened.

We calculated the percentages of stigmatizing posts to total posts by provincial area. Then, we performed repeated-measures ANOVA on these percentages to test for the changes in stigma during the different periods.

## Results

### Participants

**Fig. 2:**
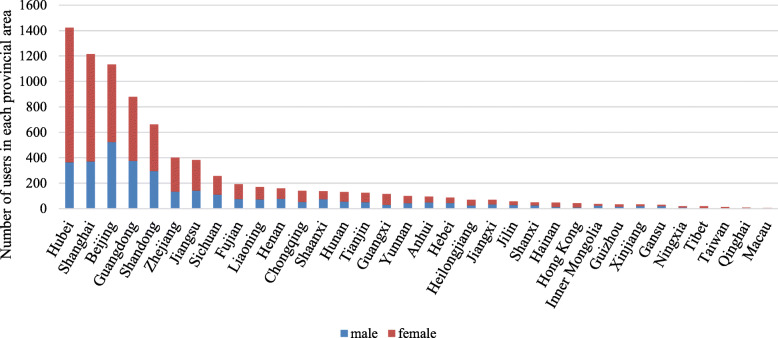
Users’ geo-locations

The total number of posts we gathered using the keywords “Wuhan people” is 19,780. 1921 posts were not related to COVID-19 and 2203 posts with users not in the provincial areas. And 15,656 posts remained after elimination, which included 8326 users. The users’ gender and location distribution are shown in Fig. 2.

### Coding

The Light’s kappa coefficients for stigmatization and attitude type were 0.76 and 0.68, respectively. The resulting kappa indicated substantial agreement between the coders on recognizing stigmatizing Weibo posts and identifying specific attitudes of stigma.

### Number of stigmatizing posts and different attitudes

The numbers of stigmatizing posts and posts that are assigned to different attitude categories are shown in Table 2**.**

**Table 2:** Proportion of Stigmatizing posts

Category	Sub-category	Number of posts	Percentage	Sum
Stigmatization	Stigmatizing	385	2.46%	15,656
	Not stigmatizing	15,271	97.54%	
Attitudes	Infectious	142	36.88%	385
	Irresponsible	116	30.13%	
	Stupid	127	32.99%	

### Daily percentages of posts of stigma and different attitude categories

**Fig. 3:**
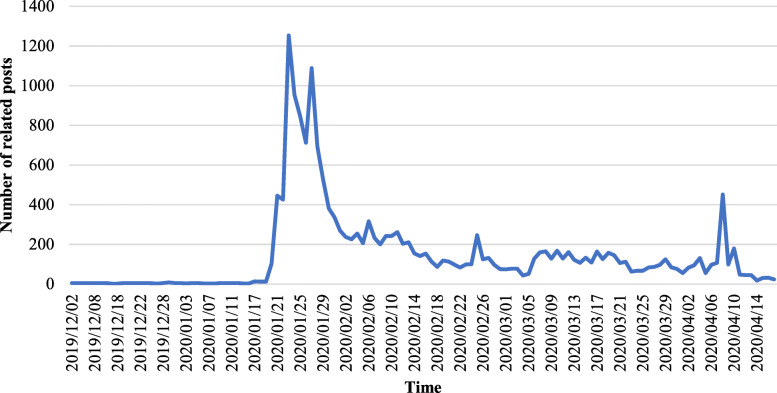
Daily number of related posts

**Fig. 4:**
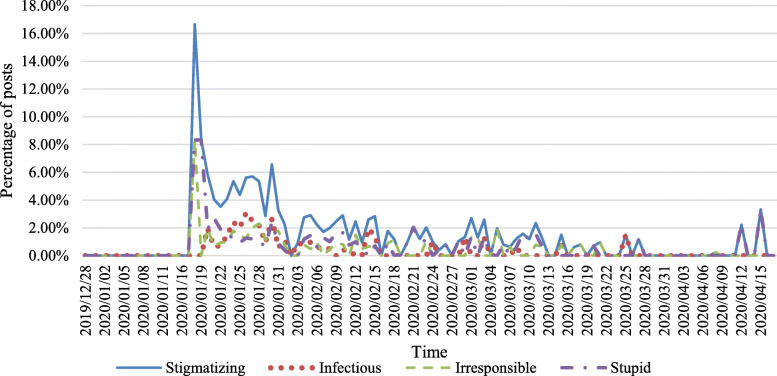
Daily percentages of posts of stigma and different attitude categories

The changes in the number of related posts over time are shown in Fig. 3. We divided the numbers of posts in each category by the number of related posts to get percentages, which are shown in Fig. 4.

In Fig. 3, the first peak point of the number of related posts is January 23, which is the day when Wuhan was lockdown (GI), and the second peak point is January 27, the day after the Wuhan government notified ‘5 million people left Wuhan before lockdown’ to explain why government intervention hadn’t taken effect (post-GI) [48]. Then related Weibo gradually declined until Wuhan was ‘unlocked’ on April 8, and the related discussions reached a small peak (eff-GI).

In Fig. 4, the percentages of stigmatizing posts reached the highest point on January 18 (pre-GI) and generally showed a downward trend afterward. Percentages of ‘Stupid’ and ‘Irresponsible’ posts rose earlier than ‘Infectious’.

### Changes of stigma in different periods

The means and standard deviations of post percentages in 34 provincial area are shown in Table 3. The percentages of stigmatizing posts differed significantly in 3 periods (F (2,66) = 5.60, *p* = .006, η^2^ = 0.15). Among the three attitude categories, percentages of ‘Infectious’ posts (F (2,66) = 3.69, *p* = .030, η^2^ = 0.10) and ‘Stupid’ posts (F (2,66) = 3.65, *p* = .031, η^2^ = 0.10) were significantly different in 3 periods. Percentages of ‘Irresponsible’ posts were not significantly different in 3 periods (F (2,66) = 0.63, *p* = .534, η^2^ = 0.02).

**Table 3:** Percentages of stigmatizing posts in three periods

Category		Period	Mean	Std. Deviation
All (Stigmatizing)		pre-GI	2.14%	4.52%
		post-GI	1.98%	2.29%
		eff-GI	0.31%	0.72%
Attitude category	Infectious	pre-GI	0.30%	1.00%
		post-GI	0.85%	1.41%
		eff-GI	0.24%	1.05%
	Irresponsible	pre-GI	1.32%	6.85%
		post-GI	0.74%	1.05%
		eff-GI	0.43%	1.92%
	Stupid	pre-GI	1.02%	2.32%
		post-GI	0.52%	0.65%
		eff-GI	0.20%	0.52%

Results of the post-hoc test show that there was no significant difference for the percentages of stigmatizing posts between pre-GI and post-GI, but a significant reduction from post-GI to eff-GI (t = − 0.017, *p* = .000). For the percentages of ‘Stupid’ posts, there was no significant difference between pre-GI and post-GI, but a significant reduction from post-GI to eff-GI (t = − 0.003, *p* = .011). For the percentages of ‘Infectious’ posts, there was a significant increase from pre-GI to post-GI (t = 0.005, *p* = .030), and a significant reduction from post-GI to eff-GI (t = − 0.006, *p* = .019).

## Discussion

This study analyzed Weibo posts that contained stigma toward Wuhan people during the COVID-19 epidemic to target specific stigmatizing attitudes and analyzed their changes for different periods of the epidemic. The results indicated that there were different attitudes associated with the stigma toward Wuhan people on Weibo, and they changed during different periods of the epidemic.

Among posts related to COVID-19, stigmatizing posts only accounted for 2.46%, which was relatively low compared with stigmatizing tweets toward the Chinese from December 31, 2019 to March 13, 2020, [21]. Nevertheless, according to Fig. 4, the percentage of stigmatizing posts reached a peak point at above 16% on Jan 18, 2020, and percentages of ‘Stupid’ and ‘Irresponsible’ posts rose earlier than ‘Infectious’. These results indicated that stigma toward Wuhan people on Weibo was relatively mild in severity from the whole period, but it was rather severe at pre-GI with ‘Irresponsible’ and ‘Stupid’ attitudes.

While the daily percentages of posts depicted subtle changes in stigma, the results from repeated-measures ANOVA gave a statistical comparison of stigma for the different periods. The main effects of the periods on stigmatizing posts, ‘Infectious’ posts, and ‘Stupid’ posts were significant, whereas for ‘Irresponsible’ posts, they were not. This suggested that government interventions may influence the stigma with ‘Infectious’ and ‘Stupid’ attitudes, but they may have no significant influence on stigma with ‘Irresponsible’ attitudes.

In the post hoc analysis, we focused on the difference from pre-GI to post-GI and from post-GI to eff-GI. Although not significant, the stigmatizing posts, ‘Stupid’ posts and ‘Irresponsible’ posts decreased from pre-GI to post-GI as shown in Table 3. In the meantime, however, ‘Infectious’ posts increased significantly. From post-GI to eff-GI, stigmatizing posts, ‘Stupid’ posts and ‘Infectious’ posts decreased significantly. These results suggested that government interventions (GI) had a negative short-term effect on ‘Infectious’ stigma but a positive long-term effect on ‘Stupid’ stigma, ‘Infectious’ stigma, and general stigma.

‘Wuhan people are infectious’ was the most common attitude associated with stigma compared with the two other types. This type of stigma is very common for other diseases [49–51]. Previous research found that Ebola-related stigma subsided as Ebola virus disease was relieved in Liberia and increased every time the disease re-emerged. In this study, we found the same consistency between the ‘Infectious’ stigma and the severity of the disease. From pre-GI to post-GI, the number of confirmed COVID-19 cases continued to increase, and ‘Infectious’ posts increased significantly. From post-GI to eff-GI, as the epidemic was under control, it decreased significantly. More importantly, government interventions, such as city lockdown and home quarantine orders, may reinforce the public’s biased perceptions about the stigmatized group without realizing it because they are following their supervisors [14]. This also explained why the ‘Infectious’ stigma increased post-GI.

The reason for the occurrence of the ‘Stupid’ stigma may be that people at pre-GI were facing a novel disease, thus speculating that the outbreak may be related to the consumption of wild animals based on the experience of SARS in the past. This form of stigmatizing attitude associated local culture with the disease and obfuscated the scientific causes of epidemics, which was also common during the SARS and H1N1 epidemics [35, 49]. At post-GI, the ‘Stupid’ stigma did not decrease significantly, indicating that popularization in the public of scientific causes of the disease was not sufficient during this period. Fortunately, ‘Stupid’ labels decreased significantly at eff-GI.

Unlike other attitudes, the ‘Irresponsible’ stigma did not decrease significantly at eff-GI. According to attribution theory [27], when people gain a strong sense of control over infectious diseases, assuming responsibility for the occurrence of disease is easy to attribute to individuals. At eff-GI, effective control of the epidemic made the public raise their expectations for individuals to avoid spreading the disease. As a result, although the number of confirmed cases decreased, people were more likely to blame the affected individuals for these cases. Thus, stigmatizing posts continued labeling Wuhan people irresponsible for escaping isolation orders.

Previous studies proved that stigma existed long after SARS [52]. With the potential re-emergence of COVID-19 any time in the future, COVID-19-related stigma could be a long-lasting problem. Our findings provide certain information for stigma mitigation. First, government interventions for disease control cannot mitigate stigma immediately. Second, stigma mitigation strategies should target specific attitudes in different periods of the epidemic. At pre-GI, a blank scientific understanding existed for a relatively long period. During this stage, stigma with ‘Stupid’ attitudes was prevalent due to the lack of scientific explanations for the disease. Target-specific strategies should correct biased explanations about the origins of the disease. At post-GI, policy-makers should consider the effects of isolation measures on stigma to avoid reinforcing stigma with ‘Infectious’ attitudes. At eff-GI, people tended to attribute the spread of the epidemic to individuals and label the individuals with ‘Irresponsible’ tags. This notion suggests that officials should explain the objective reasons for the spread of the epidemic to the public in a timely manner.

This study has certain limitations. First, attitudes toward Wuhan people online may differ from attitudes displayed offline. Second, social media users form only a sample of the Chinese population, which may not be representative of all people in China. Third, users’ demographic information was obtained from profiles, which cannot be verified. Finally, changes in different periods can only be compared over time; thus, direct causality between government interventions and stigma cannot be guaranteed.

## Conclusion

This study collected data from Weibo posts containing the keyword ‘Wuhan people’ published from December 1, 2019 to April 18, 2020, to analyze the stigma toward Wuhan people in China during the COVID-19 epidemic. This study revealed that COVID-19-related stigma in China included various attitudes and changed over time. After government interventions but before they took effect, stigma with the ‘Infectious’ attitude increased. After government interventions took effect, general stigma and stigma with ‘Infectious’ and ‘Stupid’ attitudes decreased. Our findings provide information for timely, appropriate and attitude-specific measures to be undertaken to ameliorate stigma associated with infectious diseases.

## Data Availability

The data used in the current study are available from the corresponding author on reasonable request.

## Abbreviations

COVID-19: Corona Virus Disease 2019
ANOVA: Analysis of Variance
OSN: Online Social Networks
GI: Government interventions
pre-GI: The period before government interventions
post-GI: The period after government interventions but before they took effect
eff-GI: The period after government interventions took effect.
